# War Psychiatry: Identifying and Managing the Neuropsychiatric Consequences of Armed Conflicts

**DOI:** 10.1177/21501319221106625

**Published:** 2022-06-20

**Authors:** Nityanand Jain, Sakshi Prasad, Zsófia Csenge Czárth, Swarali Yatin Chodnekar, Srinithi Mohan, Elena Savchenko, Deepkanwar Singh Panag, Andrei Tanasov, Marta Maria Betka, Emilia Platos, Dorota Świątek, Aleksandra Małgorzata Krygowska, Sofia Rozani, Mahek Srivastava, Kyriacos Evangelou, Kitija Lucija Gristina, Alina Bordeniuc, Amir Reza Akbari, Shivani Jain, Andrejs Kostiks, Aigars Reinis

**Affiliations:** 1 Riga Stradiņš University, Riga, Latvia; 2 National Pirogov Memorial Medical University, Vinnytsya, Ukraine; 3 Eötvös Loránd University, Budapest, Hungary; 4 Teaching University Geomedi LLC, Tbilisi, Georgia; 5 Charles University, Hradec Králové, Czechia; 6 Omsk State Medical University, Omsk, Russian Federation; 7 “Carol Davila” University of Medicine and Pharmacy, Bucharest, Romania; 8 Medical University of Łódź, Łódź, Poland; 9 Medical University of Warsaw, Warsaw, Poland; 10 National and Kapodistrian University of Athens, Athens, Greece; 11 “Victor Babes” University of Medicine and Pharmacy Timisoara, Timișoara, Romania; 12 Sherwood Forest Hospitals NHS Foundation Trust, Nottinghamshire, UK; 13 Genesis Institute of Dental Sciences and Research, Ferozepur, Punjab, India; 14 Riga East University Hospital, Riga, Latvia

**Keywords:** conflict, depression, neuropsychiatric effects, PTSD, treatment, stigma, refugees

## Abstract

War refugees and veterans have been known to frequently develop neuropsychiatric conditions including depression, post-traumatic stress disorder (PTSD), and anxiety disorders that tend to leave a long-lasting scar and impact their emotional response system. The shear stress, trauma, and mental breakdown from overnight displacement, family separation, and killing of friends and families cannot be described enough. Victims often require years of mental health support as they struggle with sleep difficulties, recurring memories, anxiety, grief, and anger. Everyone develops their coping mechanism which can involve dependence and long-term addiction to alcohol, drugs, violence, or gambling. The high prevalence of mental health disorders during and after the war indicates an undeniable necessity for screening those in need of treatment. For medical health professionals, it is crucial to identify such vulnerable groups who are prone to developing neuropsychiatric morbidities and associated risk factors. It is pivotal to develop and deploy effective and affordable multi-sectoral collaborative care models and therapy, which primarily depends upon family and primary care physicians in the conflict zones. Herein, we provide a brief overview regarding the identification and management of vulnerable populations, alongside discussing the challenges and possible solutions to the same.

Although several peace treaties and conventions have been agreed upon since the end of the second world war, violations, and war crimes have continued to occur across the globe.^1^ The recent Russo-Ukrainian conflict that began on the 24th of February 2022 is the latest addition to the already long list of such violations of the peace treaties. However, what makes this Russian “special military operation (SMO)” distinguishable from the ones in Iraq, Iran, Afghanistan, Vietnam, Congo, etc., is the number of civilian casualties. In each of these previous wars, defined as conflicts resulting in the deaths of at least 1000 individuals, more than 1 million people lost their lives in the cross-firing, whilst the exact numbers are not currently available for the present Russian SMO. Whilst United Nations (UN) declared 596 civilian deaths as of 10th March 2022, the Russian side has so far not acknowledged the same. Between 1945 and 2000, an estimated 41 million people have either died or were injured in multiple wars including Israel-Palestine, Afghanistan, Pakistan, Syria, Yemen, and Ukraine, amongst others.^1,2^ Interestingly, even in the 21st century, 20 of 49 reported armed conflicts in 2020, occurred in Sub-Saharan African countries.^3^

## Neuropsychiatric Illnesses in the Setting of Wars

Civilians and military personnel living in conflict and war zones have been known to frequently develop neuropsychiatric illnesses such as depression, post-traumatic stress disorder (PTSD), suicidal ideation, and anxiety disorders, which tend to leave a long-lasting scar and impact their emotional response system.^4^ The shear stress, trauma, and mental breakdown from overnight displacement, family separation, and killing of friends and families cannot be described enough. Veterans and survivors require mental health support for years after the war, as they struggle with sleep difficulties, recurring memories, anxiety, grief, and anger.^5^ All these can lead to dysfunctions in the individual coping mechanisms, often manifested in the form of over-drinking, substance abuse disorders, addiction, violence, or gambling.^6^ These mechanisms work as stress busters and motivational boosters and help the affected in socializing with their peers and family members.^6^

Specific symptoms have been observed in American veterans from the Gulf War, which led to the identification of a psychiatric syndrome called the “Gulf War Syndrome/Illness” (GWS/GWI).^7^ Veterans suffering from GWS frequently report fatigue, headache, depression, irritable bowel syndrome, panic disorder, post-traumatic stress disorder (PTSD), chronic widespread pain, and medically unexplained symptoms (MUS).^7^ Other similar functional war syndromes include shell shock, disordered action of the heart (DAH), effort syndrome, and the effects of Agent Orange. Such disorders often remain underdiagnosed due to no concrete definitions, inconsistent terminologies, and non-inclusion in ICD-10 (international classification of diseases).^8^

Hence, there is an undeniable necessity for proper screening of those vulnerable groups that are in urgent need of treatment. For medical health professionals, it is crucial to identify such groups that are prone to develop neuropsychiatric morbidities. Fourteen risk factors (grouped into 4 major categories) relating to the development of psychiatric disorders in adult war refugees have been identified in previous studies (Table 1).^4,9,10^

**Table 1.** Risk Factors in Association of Developing Depression, PTSD, and Anxiety Disorders in War-Refugees and Veterans.

Category	Risk factor	Explanation
Demographic risk factors (3)	Age	Older age increases the risk
	Gender	Females are more prone to most mental disorders except PTSD
		For PTSD both genders are equally vulnerable
	Education	A lower educational level increases the risk
War-related risk factors (2)	Number of war traumatic events	Higher the number, the higher the risk
	Conflict experience	No previous combat and prison experience increases the risk
Post-migration risk factors (7)	Duration of migration	Longer duration in exile/displaced state has been poorly but positively correlated with psychiatric disorders
	Post-migration stress	Increased stress is associated with a higher risk
	Employment	Unemployment increases risk
	Income	Lower income and savings increase the risk
	Language proficiency	Inability to comprehend the language of the host country can increase the risk
	Social support	Lack of social support increases the risk
	Marital status	Unmarried people are more susceptible to depression
		No such correlation has been established with PTSD or anxiety disorders
Other risk factors (2)	Previous neuropsychiatric disorder	Past significant medical history elevates the risk
	Child of the affected mother	Children of mothers with first-hand experience of war-related events are more vulnerable

## Identifying the Vulnerable

Identification and diagnosis of mental disorders during and after wartime is a crucial task that presents daunting challenges. Destruction of homes, livelihoods, healthcare facilities, caring for family and children, etc. forces victims to temporarily ignore their worries and anxieties. The victims are unable to process, channel, and express these feelings due to the constantly evolving situational scenarios. This leads to suppression of emotions and the potential development of neuropsychiatric conditions. Additionally, patients may not reveal or be able to recall the complete trauma experience until treatment has already started, mostly due to shame and fear.^11,12^

In terms of diagnosing neuropsychiatric conditions, there is a relative consensus amongst practitioners on how to assess common mental disorders among the victimized population and refugees. Apart from comprehensive clinical evaluation and discussion, structured questionnaires such as the HSCL-25, HTQ, and PCL, are largely employed for screening PTSD, depression, and anxiety disorders amongst war-affected populations (Table 2).^13-15^

**Table 2.** Commonly Employed Structured Questionnaires for Assessing Mental Disorders Amongst Refugees and War-Veterans.

Questionnaire	Description	Scoring/evaluation	Diagnostic purpose
The Hopkins Symptom Checklist-25 (HSCL-25)	Consists of 2 subscales—HSCL-A for anxiety (10 items) and HSCL-D for depression (15 items). Each item is scored from 1 to 4.	1. High total 25 item average correlates with the severe emotional distress of unspecified diagnosis	Assessing the existence and severity of anxiety and depression symptoms
		2. High 15-item depression average correlates with major depression (DSM-IV)^a^	
		3. Probable psychiatric case if HSCL-25 ≥1.55 whilst treatment is required if the score is ≥1.75	
Harvard Trauma Questionnaire (HTQ)	Six unique versions for different conflict refugees (Vietnamese, Cambodian, Laotian, Japanese, Croatian, and Bosnian)	In the Cambodian version, a score >2.5 is considered symptomatic of PTSD	Assessing the existence and severity of PTSD
PCL (PTSD Checklist)	The newer version comprises 20 items checklist corresponding to the DSM-V version and doesn’t have military or civilian versions. Each question is answered from 0 to 4.	In PCL-5, a provisional PTSD diagnosis can be made by treating each item rated ≥2 as symptomatic, and then following the diagnostic rule which requires at least: 1B item (questions 1-5), 1C item (questions 6-7), 2D items (questions 8-14), 2E items (questions 15-20)	A Provisional diagnosis of PTSD. The gold standard for diagnosis of PTSD is the clinically administered PTSD Scale (CAPS-5).
		A score of 31 to 33 (out of 80) is indicative of probable PTSD	
Comprehensive Trauma Inventory (CTI-104)	104 event items divided into 12 event-type scales. Each item has a check box for whether the patient experienced the event or not, followed by a 0 to 4 scale for assessing the severity of the threat/fear of that event.	Scoring can be done either by calculating the number of events experienced or the sum of the scores of the events experienced	Accessing whether the patient has experienced a traumatic event or not and if yes, evaluate the impact of the event in terms of fear and/or threat
Post Migration Living Difficulties (PMLD) Scale	23 Items scale with each item scored on a 4-point scale	Uses DSM-V scheme for diagnosis of PTSD	Assessment of current stressors amongst asylum seekers
Refugee Health Screener-15 (RHS-15)	The first 13 questions of the RHS-15 are known as the RHS-13 and relate to symptoms of depression, anxiety, and PTSD	For the RHS-13, a total score ≥11 is interpreted as a positive screening	Screening and predicting distress, anxiety, PTSD, and depression in refugees
Post-traumatic Diagnostic Scale (PDS)	12-item scale is usually administered along with HTQ. Divided into 4 parts—Part 1 for assessing exposure to a traumatic event and Parts 2 to 4 for symptoms of PTSD.	Uses DSM-V scheme for diagnosis of PTSD	Assessment of PTSD

a DSM-IV and V, diagnostic and statistical manual of the American Psychiatric Association, versions IV and V, respectively.

### Children and Minors

Unaccompanied, separated, and orphaned children are at significant risk of violence, trafficking, and sexual exploitation. These children are prone to experience separation anxiety and usually have no means of survival, or identification documents, and have witnessed shelling and open fire.^16^ They need to overcome mental challenges ranging from adjusting to a new landscape, culture, and language, to dealing with the news of their family’s demise. Another significant issue is the increasing recruitment of children in wars, assuming both ancillary and more active combat roles.^17^ Such children (often forced against will) have been shown to become vehicles of violence rather than messengers of peace for their society.^17^

Trauma and stress may be transferred from the parents to future children via subtle heritable shifts in the expression of the genome (epigenetic modulation), thereby passing along the effects for generations.^18,19^ Such trauma also increases the likelihood of developing depression, PTSD, chronic pain, migraine, heart disease, and diabetic problems as they grow.^20-22^ Night terrors and flashbacks are other common problems faced by these children. Missing schools and educational gaps are other significant issues hampering social development which cannot be handled in the short term.

The care for such minors needs to be implemented in 2 phases. Short-term targets include providing care, medical attention, stability, and most importantly letting them express their thoughts, emotions, and feel listened to. Long-term efforts that are geared toward capacity building via gradual societal integration are necessary. For child soldiers, specialized Disarmament Demobilization Reintegration Programs (DDRP) should be prioritized.^17^ It is important to stress that both targets need to be implemented in a culture and language familiar to the children. Implied and forceful introduction and adaptation would prove to be counterproductive.

Cognitive-behavioral therapy (CBT) has been recommended for child and adolescent trauma survivors.^23^ A personalized trauma-focused CBT called Teaching Recovery Techniques (TRT) should be used which includes nine 90 to 120 min sessions—7 sessions for children and 2 sessions for caregivers which are held without the children (Table 3).^24-26^ All the sessions include active skills training such as modeling, rehearsal, and homework.^26^ TRT has been shown to reduce the intensity of emotional disturbances amongst affected children.^23^ Since it is imparted in groups, TRT could prove to be logistically and financially easier to implement for the host countries.

**Table 3.** Overview of the Teaching Recovery Techniques (TRT) Sessions.

Audience—session no.	Aim of the session	Description of the session
Child—First	Introduction	Getting to know each other and identification of the major issues
Child—Second	Intrusion	Discussion about war events, news, and normalization of reactions to traumatic events. Introduction of “Safe place” visualization.
Child—Third	Intrusion	Thought discussion, use of imagery techniques, dual attention tasks, dreamwork, and distraction
Child—Fourth	Arousal	The practice of relaxation, breath control, and positive self-coping exercises. Understanding “fear thermometer” activity scheduling, and sleep hygiene.
Child—Fifth	Exposure	War event flashbacks are discussed, the concept of grading and personalized fear hierarchy is revisited, and real-life graded exposure preparation is done
Child—Sixth	Exposure	Learning about how to expose themselves to traumatic events via drawing, talking, and writing and implementation of techniques learned in the fourth session (arousal), the importance of doing enjoyable things
Child—Seventh	Follow-up	Looking into the future without discussing further the content of the intervention
Caregiver—first	Introduction	Occurs before the start of the children’s session and involves psychoeducation about traumas and how they impact children and adults
Caregiver—second	Briefing	Occurs between children’s sessions second and fourth. Caregivers are acquainted with the information that children are receiving and how caregivers can help youth to cope with past and ongoing traumas.

### Women

Women represent a neglected and often forgotten vulnerable group with an estimated 140 000 women killed each year in conflicts and wars.^27^ This neglect is explained firstly due to most women’s deaths occurring during the post-conflict period and secondly due to limited data regarding gender-specific mortality in wars.^28^ Women are at heightened risk of malnutrition, sexual abuse, poor reproductive health, and targeted killing during wars.^28^ Vitamin deficiencies and eating undercooked food have been reported to cause severe anemia and epidemics of neurodegenerative diseases including Konzo in women during conflicts.^28^ Sexual cases of abuse are frequently reported which could result in tears, abrasions, and unwanted (including ectopic and teen) pregnancies. Lack of use of protection (condoms, etc.) puts women at risk of sexually transmitted diseases (STDs). Furthermore, inadequate testing and limited treatment possibilities in a war condition can lead to long-term consequences. Pelvic inflammatory disease (PID) and associated tubal factor infertility caused by STD agents like gonorrhea or chlamydia can increase the risk of subsequent newly diagnosed bipolar disorder, depressive disorder, anxiety disorder, and sleep disorder.^29^ STDs in themselves are an equally important risk factor for depression development.^30^

A significant proportion (15%-38%) of expectant mothers require life-saving emergency and newborn care for complications that could arise during pregnancy, delivery, and immediately postpartum.^31,32^ In the context of wars, an estimated 21% of all surgeries performed are cesarean sections with an additional 6% involving other gynecological or obstetrical procedures.^33^ These women and their unborn children are at risk of higher death rates, disease mortality, congenital and developmental defects, and mental health challenges. Their pregnancy-provoked vulnerability exposes them to a greater risk of marginalization, abuse, sexual/behavioral exploitation, malnutrition, and even spontaneous abortions, which exacerbate feelings of fear, anxiety, helplessness, suicidal tendencies, or thoughts of abortion. Increased emotional stress can lead to the development of preeclampsia and gestational diabetes all of which pose serious health challenges.^34^ Psychologically there are increased risks for the development of postpartum depression and postpartum PTSD.^35^ In a situation where their partner is detained or killed in the conflict, these expectant mothers must raise their children as single parents if untoward incidents occur at home. Such single-parent women with traumatic histories have been shown to be disproportionately likely to use psychoactive substances like alcohol and tobacco.^36^

The treatment of such patients usually tends to be multifaceted. Crisis intervention programs, and group and individual therapy focused on pregnant women and their needs can undoubtedly help in mental health amelioration. The collaboration of health professionals is needed for support and treatment; gynecologists, pathologists, psychologists, psychiatrists, and nutritionists should join forces and fight against the war’s impact. Setting up safe places for childbirth, with comprehensive obstetric and neonatal care in emergency cases is necessary. Finally, it is important to use antidepressants that can be administered relatively safely in pregnancy, such as SSRIs (citalopram and sertraline).^37^ Adequate pregnancy and STD testing kits should be made available so that appropriate and timely care could be provided to women and prevent the development of long-term sequelae.

### War Veterans, Combatants, Internally Displaced, and Their Families

The traditional gender roles are still a very important part of many modern-day societies where acts of expressing emotions are viewed as signs of weakness. This leads to repression of emotions and the overall availability of men to request help regarding mental health issues. In some cases, it may lead to the development of psychiatric disorders like addiction, eating disorders, anxiety disorders, and obsessive-compulsive disorder (OCD). Depression in men involved in such conflicts often manifests in the form of increased aggressivity, irritability, and antisocial acts. For such internally displaced and combatant men, there are heightened chances of threats, kidnapping, harassment, and torture calls not only to them but also to their immediate family members. These factors increase the chances of developing cognitive problems (denial, dissociation), prolonged grief disorder, and recurrent suicidal thoughts.^38^ Whilst professional soldiers are trained to come to terms with the stressors of killing another human being, survivors’ guilt, becoming physically disabled, and difficulty readjusting to civilian life, ordinary civilians may find it impossible to cope with.

To tackle this issue, there is a need to focus on building a full spectrum of health care services including prevention, diagnostics, education, and community support. 24 × 7 hotlines need to be set up to provide counseling and emotional support. Family reintegration and therapy should be arranged for such people to better equip them to ease back into normal life. Specialized mental health screening programs for war veterans, such as the Medical Assessment Programme (MAP), the Reserves’ Mental Health Programme (RMHP), and Veterans’ Mental Health of the UK’s Ministry of Defence can be adjusted and adapted in the local context.

### Elderly

The elderly are often unable to leave conflict zones and are left alone without their family members and caregivers. Sometimes, even if they are provided the chance to leave, they refuse to do that due to their homely connection to the motherland, or for emotional reasons. The refusal mostly stems from inabilities to process the events and is a manifestation of their coping system—diving into the past or seeing a grim future. This burden of war leaves a mark on the mental state in the form of PTSD. Patients who have had PTSD in the past are clearly at risk of a relapse. According to Summers et al.,^39^ older people who were moderately or severely dependent on caregivers were more than 5 times more likely to experience serious psychological distress than those who were independent. Inaccessibility to medications, including neurotropic drugs, diabetic medications, and others compound the situation. When it is impossible to continue drug therapy, existing depressive states, and cognitive disorders tend to worsen.

Appropriate geriatric care provision is needed for the care of the elderly, which would require support from volunteers, doctors, nurses, counselors, and the government. The presence of serious disability could serve as one simple and sensitive screening criterion to be added to the screening tool used by community workers who work with the elderly.

### People Suffering From Previous Mental Disorders and Addiction

The constant exposure of addicted patients to the painful situation of war only exacerbates their sensitive condition. Patients with comorbid disorders have demonstrated poorer treatment adherence and higher rates of treatment dropout than those without mental illness, which negatively affects their overall health outcomes.^40^ Pharmacological detoxification coupled with behavioral therapy is the mainstay for treatment, maintenance of abstinence, and prevention of relapse.^40^ Buprenorphine-Naloxone, Methadone, Acamprosate, Nicotine Replacement Therapies, etc., are commonly prescribed against opioid, alcohol, and nicotine use disorders.^40^ Multiple evidence-based prevention interventions like Multisystemic Therapy (MST), Brief Strategic Family Therapy (BSFT), and Multidimensional Family Therapy (MDFT) have been shown to prevent serious antisocial behavior in addicted adolescents with substance use disorders.^40^ To manage the post-traumatic stress caused by exposure to the horrors of war, psychological, and psychiatric support from specialists specializing in both addicts and victims of war crimes is essential. Staying away from places, events, or objects that are reminders of the traumatic experience and avoiding thoughts or feelings related to the traumatic event may help in that direction. Finally, the intervention of humanitarian NGOs would be crucial both for material support (medicines, food, detoxification programs, etc.) and for the moral and psychological support of these socially vulnerable groups.

### Relatives and Friends Outside the War Zone

People who are indirectly impacted by the events of armed conflicts are also prone to developing neuropsychiatric disorders. Studies have suggested that constant exposure to graphic media images may result in physical and psychological effects.^41,42^ Relatives and friends of the war-affected people or nationals of the war-affected country that live in other countries suffer from lack of concentration, sleep deprivation, and other kinds of mental trauma. Constant exposure to social media and news outlets adds anxiety and depression to the symptomatology.^43,44^ The consumption of Covid-19-related news has contributed to anxiety issues and studies suggest people prone to such anxiety would likely seek out even more crisis coverage.^45^ This constant stress and anxiety are on a never-ending addictive wheel because stress feeds sleep deprivation and that in turn feeds stress.^45^ These people might suffer from PTSD flare-ups and physical symptoms such as high blood pressure. An early resolution of the conflict and using social and physical distractions can help divert attention. Relaxing activities and comfort food and places can relieve the symptoms.

## Challenges to Mental Healthcare System

Even though mental health issues have severely burdened the public health sector over the years, they are still not addressed with the same level of seriousness and robustness as physical health. Mental diseases are significantly underdiagnosed or misdiagnosed,^46-48^ and the patients are reluctant to seek professional support and treatment for a long time.^46,48^ Multiple challenges and shortcomings have been identified in the past that limits the accessibility and coverage of the mental healthcare system, thereby creating the so-called “mental health treatment gap.”^49,50^ Firstly, there are capacity restraints in terms of screening, case detection, service delivery, and appropriate referral to specialists. Secondly, primary health caregivers are expected to be able to screen and detect mental health problems and provide appropriate treatment and medicines. Although physicians are exposed to basic courses on psychiatry during pre-service training, many physicians report that the knowledge they obtain during formal medical education is not sufficient to provide mental health services.^51^ Thirdly, during wartime, interruptions and inadequate supplies of essential antipsychotic, antidepressant, anxiolytic, mood-stabilizing, and antiepileptic medications at mental health facilities, make it difficult to provide appropriate care. Fourthly, there is a significant lack of awareness concerning mental health issues in society and insufficient knowledge of preventive approaches and treatment possibilities.

Another significant issue is the social stigma and fear of judgment and discrimination. People traditionally have had a negative outlook on the professional efficacy and treatment of mental disorders. In the past, psychiatry has been used as a tool of repression.^52,53^ For example, people who opposed the Soviet regime were regarded as mentally ill and subjected to long imprisonments in the country’s psychiatric hospitals.^54^ As a result, the older generation in ex-Soviet countries is more reluctant to seek mental health care than the younger one, as they remember the oppressive history of the psychiatry system.^55^ Furthermore, due to high stigma and shame, people fear being labeled negatively by their communities for seeking treatment and therefore, they prefer to do so anonymously. People also fear having a public medical record that identifies them as mentally ill, as this could reduce their chances of securing employment opportunities.

For the refugees and internally displaced who were able to flee the war-affected regions, general lack of awareness regarding mental health services, discrimination, marginalization, differences in healthcare approach, language and interpretational barriers, and views of parents or relatives about the Western diagnostic paradigms remain significant issues that need to be addressed.^55,56^ Financial and logistical issues also remain to be addressed.

## Addressing the Challenges

**Figure 1.**
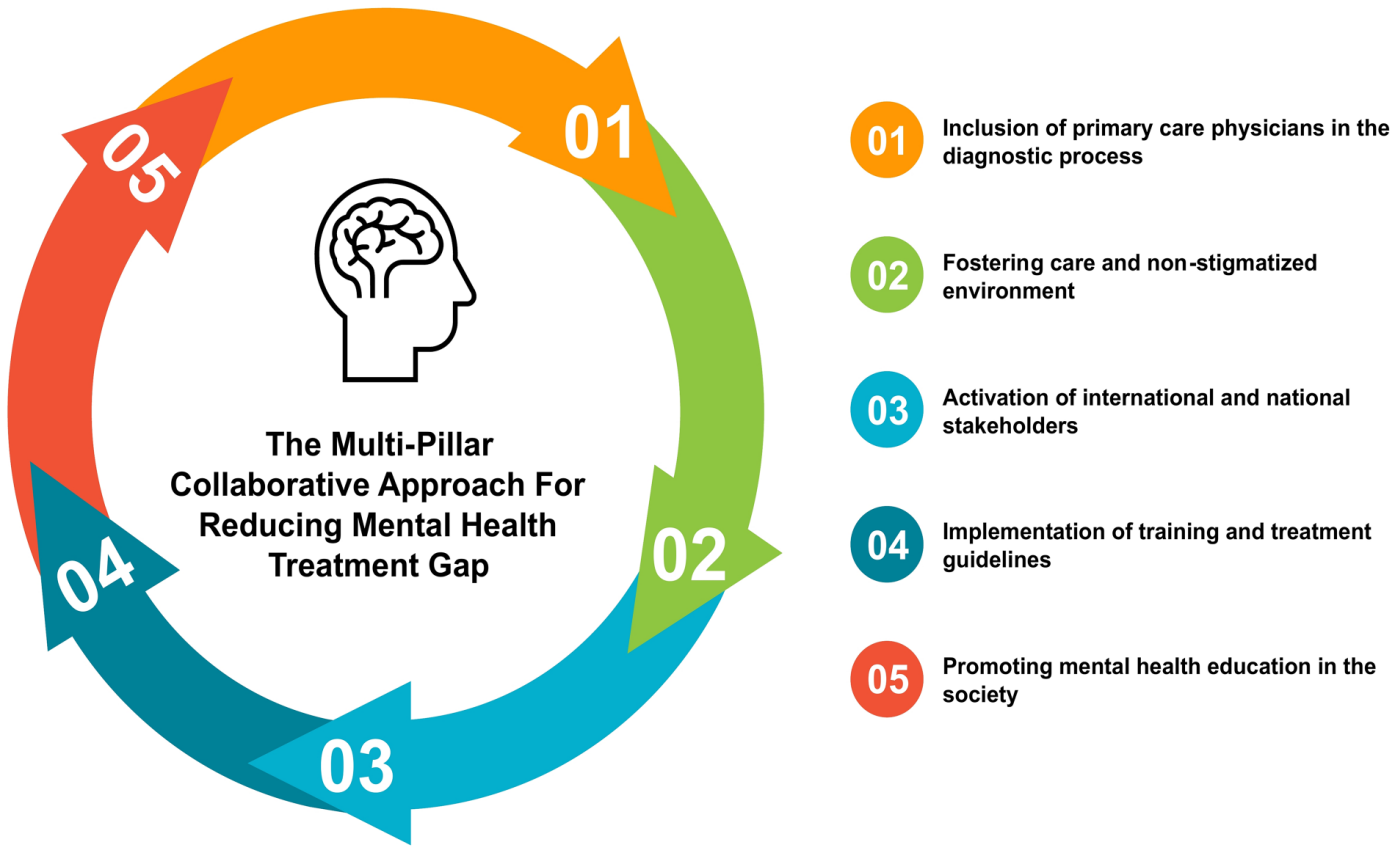
The multi-pillar collaborative approach for reducing the mental health treatment gap.

The mental health treatment gap is a serious and urgent international issue that only gets wider during armed conflicts. To address this issue, the World Health Organization (WHO) developed the Mental Health Gap Action Programme Intervention Guide (mhGAP-IG), a set of easy-to-refer clinical guidelines for providing evidence-based care.^57^ Despite its introduction, the adaptation of such guidelines remains low, especially in low- and middle-income countries.^58^ Hence, based on recommendations from previous studies, a multi-pillar collaborative approach model should be developed to address these underlying interrelated challenges and reduce the treatment gap (Figure 1).

**Pillar 1:** It is recommended to include all primary care physicians in the diagnostic process during wartime. Engel et al^59^ suggest a model which stratifies levels of care, offering a range of interventions, including preclinical prevention, symptom mitigation in routine primary care, symptom reduction and disability prevention in collaborative primary care, and intensive rehabilitation with specialist intervention only if significant disability persists.
**Pillar 2:** Fostering care and a non-stigmatized environment. It involves public collectivity and refraining from promoting inflammatory and stigmatic hate speech against the conflicted parties.^60^ The host countries should provide the incoming refugees with proper accommodation and employment opportunities along with connecting and integrating them within the community at the earliest. Special lessons on basic principles of mood and trauma management should be inculcated in the public.^60^ Making the refugees feel welcomed and at home can help them ease some of the stressors and obtain a more objective view of the situation. It is important to promote the spread of knowledge and continue anti-stigma campaigns such as “Open the Doors” (a Polish program) or “Schizophrenia Should Not Be A Reason For Discrimination” (implemented by Romanian students) to raise awareness.
**Pillar 3:** Activation of international stakeholders like the UNHCR, UNICEF, World Health Organization (WHO), International Red Cross, and Doctors without Borders (*Médecins Sans Frontières*). They should step in to provide appropriate resources and training in the absence or destruction of healthcare institutions amidst war. Healthcare workers should be given free and secure passage in the conflict zone to provide care to those in need, in line with the humanitarian principles.^61^ This is especially important since, in the previous conflicts, a mass efflux of doctors early in the conflict has been recorded due to the targeted threats, and increased risks of kidnapping or death (also called irregular violence).^62,63^
**Pillar 4:** Another important pillar in this regard is the appropriate training and familiarity of the doctors to be situation-ready for such a crisis. Whilst there are exposure opportunities in the form of conferences, seminars, simulations, and hands-on courses, they have multiple shortcomings including lack of integration with general mental health training, absence of outcome assessments, a generalized approach rather than a situation-specific approach, and questionable relevancy.^64,65^ To address these challenges, there are 2 possible ways. The first one includes appropriate training given to medical students during medical school. The second one is the implementation of ISTSS/Rand Guidelines on Mental Health Training of Primary Healthcare Providers for Trauma Exposed Populations in Conflict-Affected Countries which ensures that primary responders provide high-quality mental health care to the affected.^66^
**Pillar 5:** The final pillar is promoting mental health education regarding its diagnosis, treatment possibilities, and preventive measures. Appropriate social media channels, online posters, books, pamphlets, announcements, TV adverts, etc., should be made available immediately.

## Conclusions

The victimized population constitutes a largely heterogeneous group with different backgrounds, medical history, experiences, and ways of coping, which makes it difficult to attempt a personalized approach to identifying and treating those in need of help. The major barriers to access to mental health care in war-affected regions include lack of trust in the psychiatry system, stigma and shame, and lack of awareness and understanding. Identification of risk factors and vulnerable population groups is essential for providing early and timely interventions. Interventions should be aimed at preventing the long-term sequelae of the symptoms and requires a multi-pillar collaboration model for achieving maximum coverage. It is pivotal to develop and deploy effective and affordable multi-sectoral collaborative care models and therapy, which primarily depends upon family and primary care physicians in conflict zones.
